# Aversive Reinforcement Improves Visual Discrimination Learning in Free-Flying Honeybees

**DOI:** 10.1371/journal.pone.0015370

**Published:** 2010-10-15

**Authors:** Aurore Avarguès-Weber, Maria G. de Brito Sanchez, Martin Giurfa, Adrian G. Dyer

**Affiliations:** 1 Université de Toulouse, UPS, Centre de Recherches sur la Cognition Animale, Toulouse, France; 2 CNRS, Centre de Recherches sur la Cognition Animale, Toulouse, France; 3 Department of Physiology, Monash University, Clayton, Victoria, Australia; Université Pierre et Marie Curie, France

## Abstract

**Background:**

Learning and perception of visual stimuli by free-flying honeybees has been shown to vary dramatically depending on the way insects are trained. Fine color discrimination is achieved when both a target and a distractor are present during training (differential conditioning), whilst if the same target is learnt in isolation (absolute conditioning), discrimination is coarse and limited to perceptually dissimilar alternatives. Another way to potentially enhance discrimination is to increase the penalty associated with the distractor. Here we studied whether coupling the distractor with a highly concentrated quinine solution improves color discrimination of both similar and dissimilar colors by free-flying honeybees. As we assumed that quinine acts as an aversive stimulus, we analyzed whether aversion, if any, is based on an aversive sensory input at the gustatory level or on a post-ingestional malaise following quinine feeding.

**Methodology/Principal Findings:**

We show that the presence of a highly concentrated quinine solution (60 mM) acts as an aversive reinforcer promoting rejection of the target associated with it, and improving discrimination of perceptually similar stimuli but not of dissimilar stimuli. Free-flying bees did not use remote cues to detect the presence of quinine solution; the aversive effect exerted by this substance was mediated via a gustatory input, i.e. via a distasteful sensory experience, rather than via a post-ingestional malaise.

**Conclusion:**

The present study supports the hypothesis that aversion conditioning is important for understanding how and what animals perceive and learn. By using this form of conditioning coupled with appetitive conditioning in the framework of a differential conditioning procedure, it is possible to uncover discrimination capabilities that may remain otherwise unsuspected. We show, therefore, that visual discrimination is not an absolute phenomenon but can be modulated by experience.

## Introduction

The honeybee is a useful model for the study of complex visual-problem solving by a miniature brain [1]–[3]. Despite their relative small brain, honeybees learn to navigate mazes [4], use top-down processing to break camouflage [5], solve delayed matching to sample tasks and thus demonstrate rule learning [6], process rotated complex objects like faces [7], categorize complex spatial information [8]–[10] and exhibit performances akin to numerical counting [11]–[12].

A crucial aspect to reveal the cognitive capacities of honeybees, and other animals, is the training procedure employed by the experimenter. For instance, in several cases of complex problem solving by bees, learning sets have been used in which insects were confronted with a random succession of changing stimuli in which a specific feature remained constant and associated with reward. In this way, it was possible to ask whether bees are able to extract this feature *per se* and solve a problem on its basis (e.g. symmetry categorization [8]; orientation categorization [13]; configurational categorization [9]–[10]). Recent work has also demonstrated that the learning of color stimuli for both bumblebees [14] and honeybees [15] is dependent on the type of conditioning procedure. Specifically, fine color discrimination is achieved when both a target (rewarded conditioned stimulus or CS+) and a distractor (non-rewarded conditioned stimulus or CS−) are present during training (differential conditioning), whilst if the target is learnt in isolation (absolute conditioning), discrimination is coarse and limited to perceptually dissimilar alternatives. Thus, differential conditioning procedures seem to promote high levels of visual discrimination. An explanation provided to account for differences in discrimination resulting from absolute and differential conditioning is the hypothesis that the former, contrary to the latter, recruits attentional processes that are necessary to learn the difference between a target and a distractor [15].

Different procedures allow enhancing attention levels during differential conditioning. Manipulation of reinforcer intensity and/or hedonic value is a possible strategy. In any differential conditioning two specific memory traces are established, an excitatory one derived from CS+ experiences, and an inhibitory one derived from CS− experiences; choice following conditioning with a CS+ and a CS− results from the interaction between these two traces [16]. Thus, increasing the penalty associated with a CS− could enhance discrimination performances. In the case of free-flying bees subjected to visual discrimination problems, traditional differential conditioning procedures reward the CS+ with sucrose solution (usually in a range between 30% and 50% weight/weight) and do not reward the CS−. In this experimental framework, one could ask whether coupling the CS− with an aversive reinforcement (instead of presenting it without reinforcement) could further improve learning performance in honeybees. To increase the penalty associated with a CS−, and to potentiate learning abilities of free-flying bees, we decided to pair the visual stimulus acting as CS− with a highly concentrated quinine solution.

The choice of quinine was based on recent studies reporting that quinine promotes accurate learning of stimuli in bumblebees [17]–[20]. These studies, however, contrast with the fact that naturally occurring concentrations of secondary compounds in nectar that taste bitter to human do not have a deterring effect in free-flying honeybees but can even elicit a feeding preference [21]. Moreover, to date there has been no evidence of bitter gustatory receptors being present in the honeybee (electrophysiology [22] or genomic study [23]; see [24] for review). Additionally, experiments in the laboratory with harnessed bees could not find clear evidence supporting that bitter compounds are aversive to bees in contention [22], [25].

Given this apparent lack of agreement on the aversive nature of bitter compounds for honeybees, we decided to explicitly test whether a highly concentrated quinine solution would confer an enhanced aversive associative strength to a visual CS−, thus facilitating its discrimination from a CS+. In asking this question we took into account the perceptual similarity of the visual stimuli to be discriminated and analyzed whether the negative reinforcement would indistinctly favor discrimination both for dissimilar and similar stimuli. To understand the mechanisms underlying behavioral performances, we analyzed whether quinine aversion, if any, is based on an aversive sensory input at the gustatory level or on a post-ingestional malaise following quinine feeding.

## Results

### Experiment 1: Does quinine improve visual discrimination of perceptually dissimilar colors when used as negative reinforcer?

**Figure 1:**
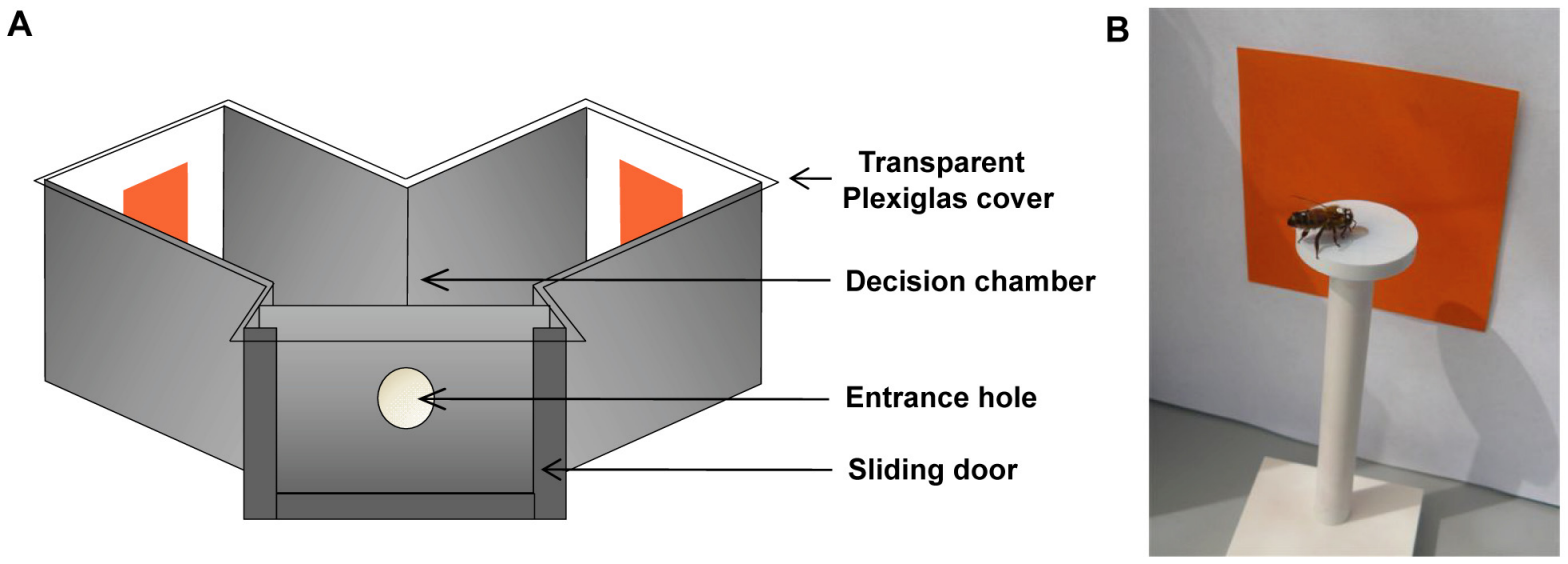
Set-up used in this study. (A) Diagram of the Y-maze used in the experiments. (B) Photograph showing how stimuli were presented in association with a solution holder in each arm of the Y-maze during experiment 3.

The potential aversive effect of quinine leading to an improvement of visual discrimination was investigated using a differential conditioning protocol in which one color stimulus (CS+) was associated with sucrose solution 1 M and another color (CS−) with either a highly concentrated 60 mM quinine solution (quinine group) or tap water (water group). Colors were presented in a Y-maze (Fig. 1a) to which honeybees were individually trained. Only one marked bee was present at a time in the Y-maze.

**Figure 2:**
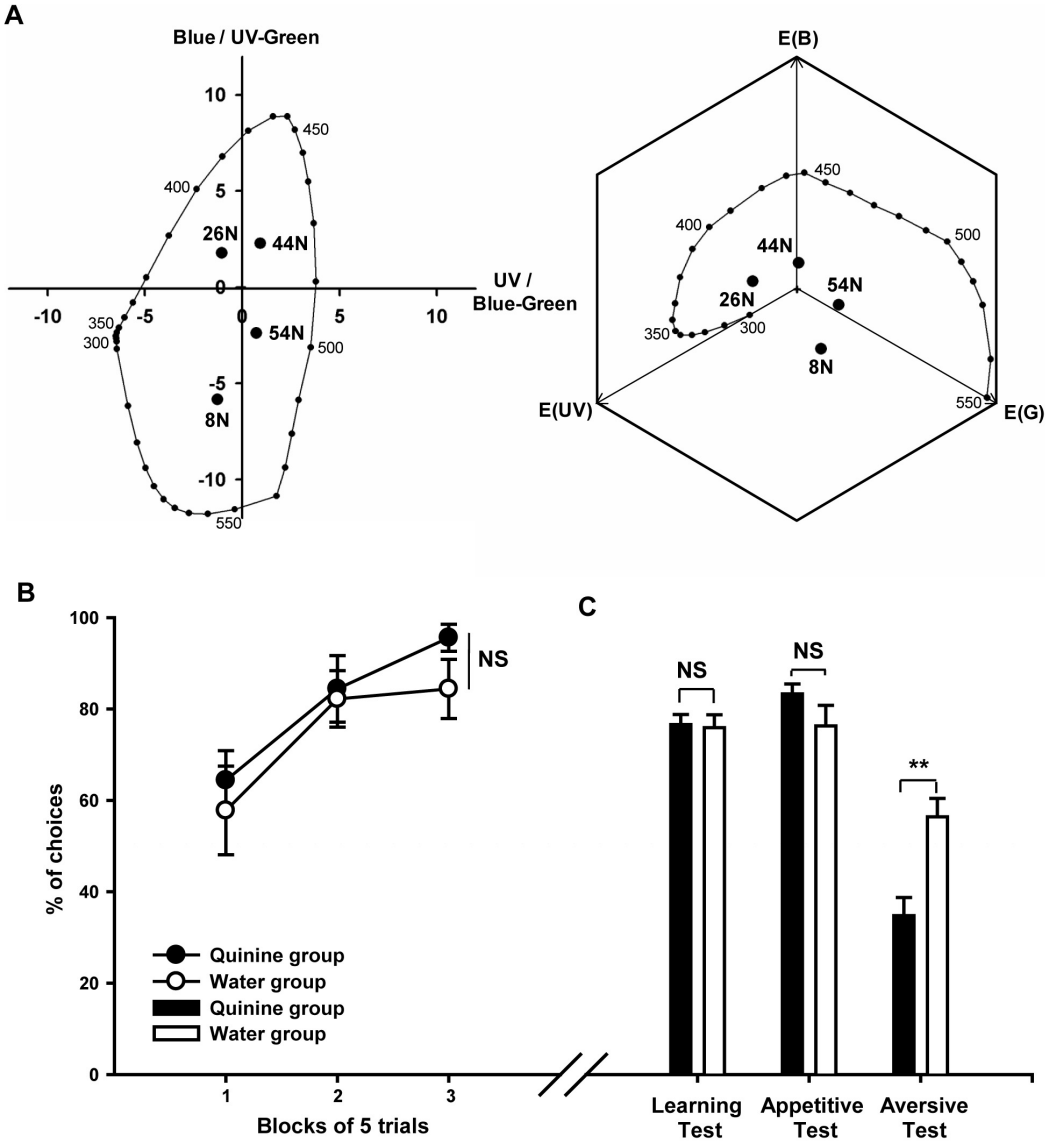
Results of Experiment 1: Does quinine improve visual discrimination of perceptually dissimilar colors when used as negative reinforcer? (A) Plots of colored stimuli used in experiment 1 in a COC color space (left) and hexagon color space (right) for the trichromatic vision of honeybees. The colour distances between stimuli are above 2.5 COC units and above 0.2 hexagon units. Numbers refer to the HKS papers references. (B) Learning acquisition (correct choices (%) by blocks of 5 trials; means ± s.e.m.; N = 9 for each curve). The curve with black dots represents acquisition by the quinine group (CS− reinforced with quinine); the curve with white dots represents acquisition by the water group (CS− reinforced with water). (C) Performance (means + s.e.m. of percentages of CS+ choices (‘learning’ and ‘appetitive’ test) or CS− choices (‘aversive’ test); N = 9 for each bar) in non-rewarded tests. Black bars represent the results of the quinine group; white bars represent the results of the water group. The learning performance in this easy colour discrimination task was not significantly different between test groups. Bees from the quinine groups avoided the stimulus associated with quinine during training when proposed versus a neutral stimulus, however this avoidance was not found in the water group (**: p<0.005).

Color stimuli were cut from broadband HKS-N colored papers. Four colors were used in this experiment (HKS 8N, 26N, 44N and 54N, which appeared orange, pink, blue and green to humans respectively; see Fig. 2a). All six dual combinations were used as conditioning stimuli (8N vs. 26N, 8N vs. 44N, 8N vs. 54N, 26N vs. 44N, 26N vs. 54N, 44N vs. 54N). Bees in the quinine group and in the water group were matched with respect to its training combination. All colors were easily distinguishable for bees as they were well separated from each other irrespective of the color space used to represent them *(color opponent coding space*: mean perceptual distance between stimuli ± s.e.m. = 6.15±1.10 COC units; *hexagon color space*: 0.31±0.04 hexagon units).

Each bee was trained with its particular CS+ vs. CS− combination for 15 trials. Afterwards, it was subjected to three different non-rewarded tests: a learning test presenting the CS+ vs. the CS−; a test presenting the CS+ vs. a novel stimulus (NS) (‘appetitive’ test), and a test presenting the CS− vs. NS (‘aversive’ test). Whilst the learning test allows verifying whether or not bees learned the discrimination task, the CS+ vs. NS test verified that the CS+ gained an excitatory associative strength leading the bees to choose it preferentially; the CS− vs. NS test, on the contrary, assesses whether or not the CS− has gained an inhibitory associative strength, leading the bees to avoid it and to prefer the NS. For each bee trained with a particular combination of colors, one of the two remaining colors not used during the training was assigned randomly as NS for the tests.

Both groups of bees (quinine group and water group) learned the task as their acquisition curves significantly increased during the three blocks of 5 trials (Fig. 2b; ANOVA for repeated measurements; N = 18 bees; block effect: F_2,32_ = 12.7, p<0.001). There was no group effect (F_1,16_ = 1.8, p = 0.20) thus showing that, at least at the level of acquisition, having quinine or water associated with the CS− did not significantly affect discrimination learning.

In the learning test, bees of both groups preferred the CS+ to the CS− (quinine group: N = 9 bees; 82.3±2.7% correct choices; mean ± s.e.m., black bar in Fig. 2c ‘learning test’; water group: N = 9 bees; 75.9±2.8%, white bar in Fig. 2c ‘learning test’). In both cases, the percentage of correct choices differed significantly from a random choice (quinine group: one-sample t-test against 50%; t_8_ = 9.7, p<0.001; water group: t_8_ = 7.7, p<0.001), thus confirming that both groups learned the discrimination between CS+ and CS−. There were no significant differences between groups (two-sample t-test: t_16_ = 1.6, p = 0.14).

In the test comparing the CS+ and the novel stimulus (NS), both groups of bees preferred the CS+ to the NS (quinine group: 76.6±2.2% correct choices, t_8_ = 11.2, p<0.001, black bar in Fig. 2c ‘appetitive test’; water group: 76.3±4.5%, t_8_ = 5.4, p<0.005, white bar in Fig. 2c ‘appetitive test’). There were no significant differences between groups (two-sample t-test: t_16_ = 0.1, p = 0.90), which shows that the CS+ gained a similar excitatory strength in both cases, a result that was expected given that the same appetitive US was paired with the CS+ for both groups of bees.

Differences between groups were evident in the test comapring the CS− and the NS. The quinine group avoided the CS− and chose therefore the NS (34.8±4.0% of choices for CS−; t_8_ = 3.8, p<0.01; black bar in Fig. 2c ‘aversive test’) while the water group did not avoid the CS− (56.4±4.0% of choices for CS−; t_8_ = 1.6, p = 0.16, white bar in Fig. 2c ‘aversive test’). The performance of both groups was significantly different (t_16_ = 3.8, p<0.005), indicating that only quinine induced an aversion of the CS− in free-flying honeybees.

We analyzed whether performance in the tests was not affected by color-specific effects so that choices were independent of the particular color distance separating the test stimuli presented to each bee. This analysis is important because the basic assumption of this experiment is that given the clear dissimilarity between the colors used, the performance of the bees would be similar in all cases. To test this assumption we performed Pearson correlation analyses between the percentage of choices for a CS (CS+ in the learning and appetitive tests, and CS− in the aversive test) and the color distance between the stimuli presented in each test. For the tests in which there was no difference between the performance of the quinine and the water groups (learning and appetitive tests; see above), data from both groups were pooled. For the aversive test, both groups were treated separately as performances in the aversive test were significantly different. Distances used in the correlation analyses were derived from the COC and the hexagon colour models (independent analyses).

When considering all color combinations used, performance in the learning test confronting the CS+ and the CS− was independent of the color distance separating these two stimuli (COC: r = 0.1578, p = 0.53; hexagon: r = −0.0337, p = 0.89). The same result was obtained for the appetitive test confronting the CS+ and the NS (COC: r = −0.0911, p = 0.72; hexagon: r = −0.2872, p = 0.25). Finally, in the aversive test confronting the CS− and the NS, choices were independent of the distance separating these stimuli both for the quinine (COC: r = −0.1852, p = 0.63; hexagon: r = −0.2272, p = 0.56) and the water group (COC: r = −0.5619, p = 0.12; hexagon: r = −0.6434, p = 0.06). These results show, therefore, that performance was not affected by differences in color distances, which were all large enough as to facilitate discrimination.

Based on these results, the large perceptual distance between color stimuli is a potential explanation why we did not observe significant differences in acquisition between the quinine and the water groups (see Fig. 2b and 2c, ‘learning test’). Specifically, as discrimination was facilitated by the dissimilarity of colors, using quinine may not have lead to an improvement in performance due to a ceiling effect in the water group. Hence in experiment 2 we trained bees in a differential conditioning protocol using colors that were perceptually closer and thus more difficult to discriminate.

### Experiment 2: Does quinine improve visual discrimination of perceptually similar colors when used as negative reinforcer?

We used the method described for experiment 1, but using perceptually similar visual stimuli as CS+ and CS−. Our goal was to determine if with increased task difficulty quinine would improve visual discrimination due to its potential aversive nature. In this experiment, only the learning test was performed after conditioning, as we aimed at detecting potential differences in learning of a difficult visual task.

**Figure 3:**
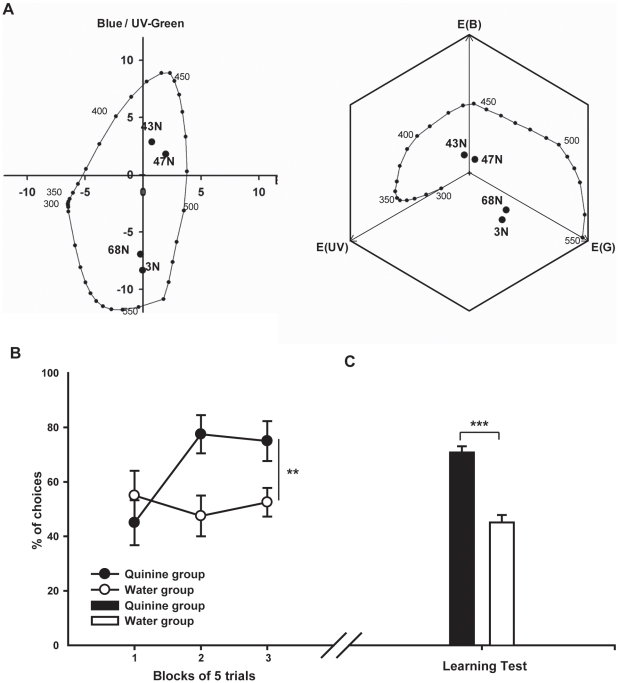
Results of Experiment 2: Does quinine improve visual discrimination of perceptually similar colors when used as negative reinforcer? (A) Plots of colored stimuli used in experiment 2 in a COC color space (left) and in a hexagon color space (right) for the trichromatic vision of honeybees. Distances between the two stimuli used for each bee are 1.5 COC units and 0.08 hexagon units for the ‘yellow’ group (3N vs. 68N) and 1.3 COC units and 0.08 hexagon units for the ‘blue’ group (43N vs. 47N). Numbers refer to the HKS papers references. (B) Learning acquisition (% of correct choices by blocks of 5 trials; means ± s.e.m.; N = 8 for each curve). The curve with black dots represents acquisition by the quinine group (CS− reinforced with quinine); the curve with white dots represents acquisition by the water group (CS− reinforced with water). (C) Performance (means + s.e.m. of correct choices; N = 8 for each bar) in non-rewarded learning test. The black bar represents the results of the quinine group; the white bar represents the results of the water group. Only bees from the quinine group solved this difficult discrimination task (**: p<0.005; ***: p<0.001).

Four colors were used in this experiment (HKS 3N, 43N, 47N and 68N), two of which (3N and 68N) appeared yellow to humans, and the other two blue (43N and 47N) (see Fig. 3a). Bees were trained to discriminate the similar colors (i.e. either 3N vs. 68N or 43N vs. 47N), which, irrespective of the color space used to represent them, were perceptually close *(color opponent coding space*: mean perceptual distance between stimuli ± s.e.m.  = 1.47±0.11 COC units; *hexagon color space*: 0.08±0.003 hexagon units).

A clear difference between the quinine and the water group was apparent during acquisition (Fig. 3b). While bees from the quinine group learned the discrimination (N = 8 bees; F_2,12_ = 4.1, p<0.05, black dots in Fig. 3b), bees from the water group did not learn to discriminate the CS+ from the CS− (N = 8 bees; F_2,12_ = 0.3, p = 0.78, white dots in Fig. 3b). The difference between groups was significant (group effect, F_1,14_ = 10.1, p<0.01; group × trial interaction, F_2,28_ = 3.6, p<0.05), thus showing that for a perceptually difficult color discrimination, associating quinine with the CS− facilitates discrimination by free-flying honeybees. This conclusion was confirmed by the results of the learning test, in which bees of both groups were again confronted with the CS+ and the CS− in the absence of reinforcement. Whilst bees in the quinine group significantly preferred the CS+ (70.8±2.3% of correct choices, t_7_ = 8.6, p<0.001; black bar in Fig. 3c), bees in the water group did not discriminate between CS+ and CS− (45.1±2.7% of correct choices, t_7_ = 1.8, p = 0.12; white bar in Fig. 3c). The difference between groups was highly significant (t_14_ = 7.1, p<0.001).

We analyzed again whether color specific effects affected test performance of bees. Two color distances were used for the Pearson correlation analyses (those between 3N and 68N and between 43N vs. 47N). A significant correlation between color distance and correct choices for the CS+ was neither found for the quinine group (COC: r = −0.3965, p = 0.33; hexagon: r = 0.3965, p = 0.33) nor for the water group (COC: r = 0.3696, p = 0.37; hexagon: r = 0.3696, p = 0.37). This result thus shows that no color specific effects affected the bees' performance. When the visual discrimination that has to be achieved by freely-flying bees is perceptually difficult, a highly concentrated quinine solution (60 mM) facilitates discrimination acting as an effective negative reinforcer.

### Experiment 3: Possible mechanisms accounting for the aversive nature of quinine solution

The highly concentrated quinine solution could exert its aversive effect via different physiological processes. Two plausible options would locate the aversive effect either at the periphery, i.e. at the level of gustatory receptors, or at a general internal, physiological level. While the former option would consist in a distasteful gustatory experience elicited by quinine solution, the latter option would consist of a post-ingestional malaise induced by the quinine solution, which would not necessarily taste bad to bees but which would be toxic once ingested [25].

To determine the process by which quinine solution exerts its aversive effect, we designed an experiment to compare the quantities of quinine solution and water imbibed by bees in similar experimental conditions. While gustatory aversion would be consistent with bees imbibing significantly less quinine solution than water, post-ingestional malaise would be consistent with bees imbibing comparable volumes of water and quinine solution. In the latter scenario, only after ingestion would bees experience the malaise effect and thus the aversive nature of quinine solution.

Bees were trained to collect sucrose solution in the Y-maze. They faced an impossible task as the same visual stimulus was presented in both arms of the maze during 15 trials. Half of the bees were trained with HKS 8N (orange to humans) and the other half with HKS 54N (green to humans). During the first 5 trials the color chosen for training was rewarded with 35 µL of sucrose 1 M (‘target’) whilst the same stimulus on the other side contained no reinforcement (‘distractor’). After the first 5 trials, for one group of bees the ‘distractor’ was associated with 35 µL of quinine solution 60 mM from trials 6 to 10 and with 35 µL of water from trials 11 to 15; for a second group of bees the sequence was inverted, so that water was obtained on the distractor from trials 6 to 10 and quinine solution from trials 11 to 15.

**Figure 4:**
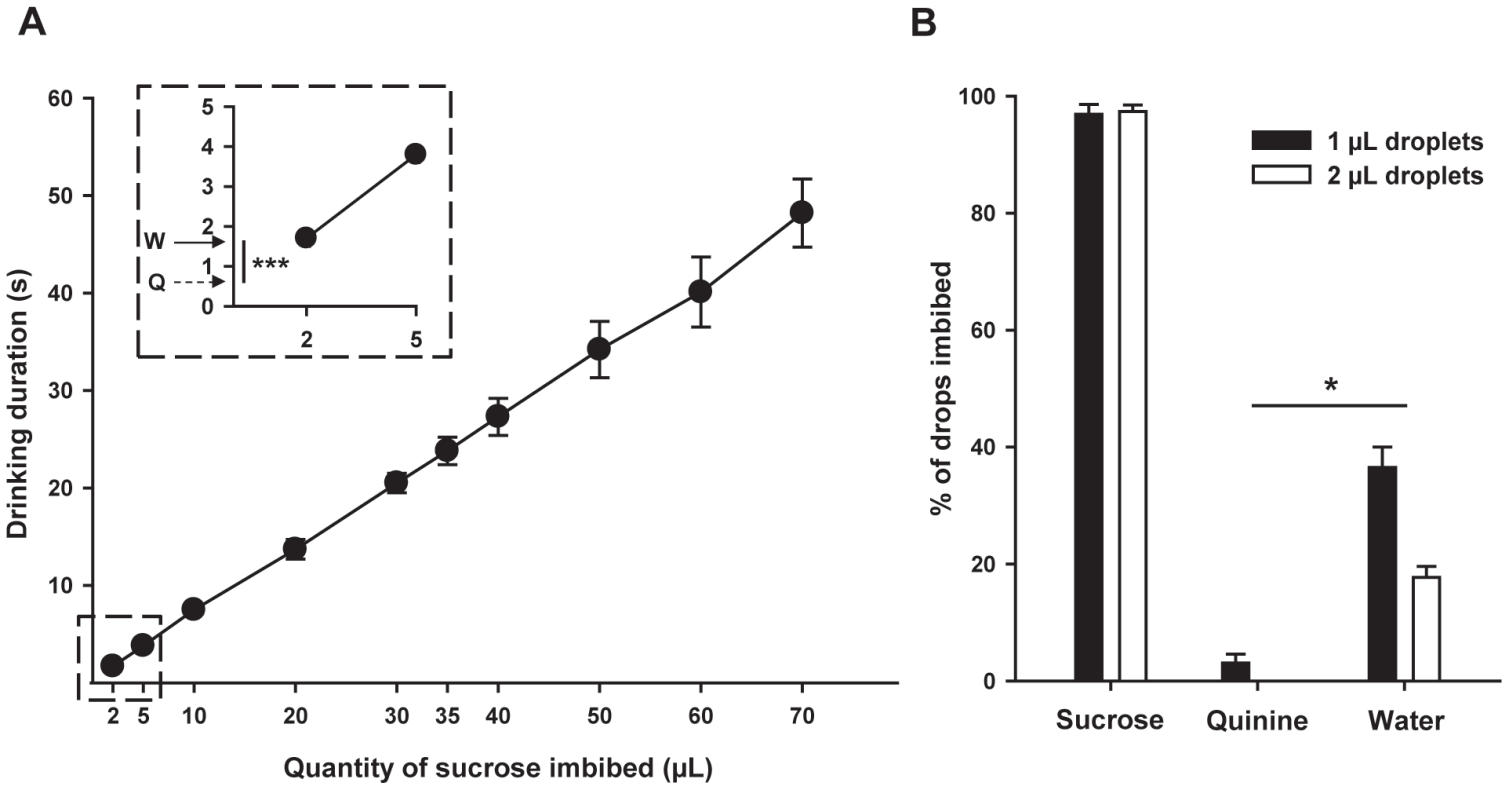
Results of Experiment 3: Possible mechanisms accounting for the aversive nature of quinine solution. (A) Standard curve relating drinking duration (s) and volume of 1 M sucrose solution imbibed (µL). These variables were linearly correlated (p<0.001). The arrows in inset show the means of water (W) and quinine (Q) drinking duration (***: p<0.001). (B) Percentage of droplets (1 µL in black or 2 µL in white) of sucrose, quinine or water totally imbibed by bees when presented together on a plate. These data show that in a foraging context, free-flying honeybees don't imbibe quinine but do imbibe water (*: p<0.05).

In order to estimate the volume imbibed by each bee, we established a standard curve relating drinking duration (sec) and volume of 1 M sucrose solution imbibed (µL). The relationship between both variables was almost linear (0.7 µL/sec; Pearson linear correlation analysis: r = 0.9997, p<0.001; N = 8 bees; Fig. 4a), and is consistent with previous estimations obtained by Núñez [26].

During the three blocks, bees made random choices between the ‘target’ and the ‘distractor’, a result that was expected given that the same stimulus was used for both categories. There was no block effect (F_2,12_ = 2.7, p = 0.11), showing that irrespective of ‘distractor’ reinforcement (quinine, water or nothing), the percentage of choices of the ‘target’ remained the same. Furthermore, the sequence of presentation of negative reinforcements (nothing, water and quinine vs. nothing, quinine and water; see above) did not affect the percentage of choices of the ‘target’ (F_1,6_ = 0.6, p = 0.46). These results show that the sequence of presentation of quinine and water did not affect performance. The results also show that the different solutions were not remotely detected by olfactory cues. A similar analysis was performed on the time spent by bees drinking the negative reinforcer associated with the ‘distractor’ (quinine or water). As the experiment included a block of trials in which there was no reinforcer on the ‘distractor’, drinking time values assigned to this block was zero in all cases. As in the previous analysis, the sequence of presentation of negative reinforcements (nothing, water and quinine vs. nothing, quinine and water; see above) did not affect drinking time (F_1,6_ = 0.74, p = 0.42). However, the block effect was significant (F_2,12_ = 59.4, p<0.001), showing that drinking time varied with the type of reinforcement provided. In fact, post hoc Tukey tests showed that bees spent significantly less time drinking quinine than water (mean of 5 trials: water: 1.7±0.3 sec; quinine: 0.7±0.2 sec; p<0.001; see inset in Fig. 4a). This result shows that bees only consumed very low volumes of quinine, if any. Indeed, such a short time may be just enough for the bee to extend the proboscis and taste the solution, and then reject it without significant drinking. Hence drinking time values indicate that the aversive effect of quinine was not determined by a post-ingestional malaise but rather by a distasteful gustatory experience.

As the mean drinking times for quinine solution and water correspond to theoretical volumes (0.7 µL and 1.7 µL) that are below the initial point (2 µL) of our time-volume curve (see inset in Fig. 4a), we performed an additional experiment to more precisely quantify the imbibing activity of free-flying bees confronted with 16 drops of 1 and 2 µL of 1 M sucrose solution, water or highly concentrated quinine solution (60 mM). Drops were randomly placed on a Plexiglas sheet housed within box with a UV transparent Plexiglas cover. During 6 visits to the setup, each lasting 180 sec, we quantified for each substance the number of drops that were consumed. In visits 1, 3 and 6, bees were presented with 8 drops of sucrose and 8 of water while in visits 2, 4 and 5, they were presented with 8 drops of sucrose and 8 of quinine solution.

Figure 4b shows that bees imbibed nearly all presented sucrose droplets (N = 8 bees, 96.9±1.7% of 1 µL droplets, 97.4±1.1% of 2 µL droplets), and a reasonable number of the water droplets (36.5±3.5% of 1 µL droplets, 17.7±1.9% of 2 µL droplets). However, they almost never imbibed the quinine droplets (3.1±1.5% of 1 µL droplets, 0.0±0.0% of 2 µL droplets). Both for the 1 µL and the 2 µL droplets, there was a significant difference between the quantity of water and quinine droplets imbibed (1 µL droplets: Wilcoxon sign ranks test; Z = 2.52, p<0.05; 2 µL droplets: Z = 2.52, p<0.05).

One possibility for the very low frequency of bees imbibing quinine could be that they used olfaction to detect and avoid quinine droplets at a very close range, even if we showed that this was not the case in the Y-maze experiment (see above). To test this possibility the proportion of droplets tasted by bees (droplets for which proboscis extension was observed) was evaluated (number of droplets tasted/total number of droplets). The proportion of tasted droplets was similar for each solution (98.7±0.5% of sucrose droplets, 97.9±1.2% of water droplets and 97.9±0.9% of quinine droplets). A similar result was obtained even if data from the first trial were not taken into account to exclude presentation order effects. Thus, consistent with the preceding experiment in the Y-maze, bees showed no evidence of sensing the presence of quinine prior to tasting it via their proboscis.

These data show that in a foraging context, free-flying honeybees do not imbibe quinine, even on their first encounter, which suggests an ability to taste quinine and to label it as an aversive substance via a gustatory input. The aversive effect induced by this substance in visual discrimination experiments would be based on a distasteful gustatory experience rather than on a post-ingestional malaise.

## Discussion

The present work shows that visual discrimination by free-flying bees is not an absolute phenomenon but a process that can be modulated in a significant way by the nature of reinforcers associated with the visual stimuli that have to be discriminated. In our work we show that the presence of a highly concentrated quinine solution (60 mM) acts as an aversive reinforcer promoting rejection of the target associated with it, and improving discrimination in perceptually difficult tasks (discrimination of perceptually similar stimuli). We show that bees do not use remote cues to detect the presence of the quinine solution and that the aversive effect exerted by this substance, in the case of free-flying bees, is mediated via a gustatory input, i.e. via a distasteful sensory experience, rather than via a post-ingestional malaise.

### Improving visual discrimination by means of a negative reinforcer

Colour discrimination was usually described as a fast form of learning [27], compared, for instance, with learning of visual patterns, which usually takes longer (twenty or more trials). Recent studies on bumblebee and honeybee color and pattern learning [14]–[15], [28] have introduced a new view of visual learning by free-flying bees, by showing that what the insects learn and discriminate in a given visual task depends on the training procedure which may or not inculcate the use of specific cues for solving a discrimination problem. It was previously thought that what an animal sees and visually learns is constrained by its perceptual machinery with little or no place for experience-dependent modulations of perception. The studies mentioned above and our work show that this idea is wrong: in some cases, learning a particular color may occur after few trials, but in other cases with perceptually similar stimuli it may need many more trials incorporating aversive conditioning.

Previous work has shown that bees [14]–[15] and ants [29] exhibit different visual discrimination powers after absolute conditioning (promoting less discrimination power) and differential conditioning (promoting more discrimination power) of color stimuli. Here we show that a negative reinforcer associated with a distractor in differential conditioning significantly enhances visual discrimination power. Visual discrimination in free-flying bees is usually studied in protocols in which one target is rewarded with concentrated sucrose solution (usually 1 M) and one or various distractors are presented without any reinforcement. In the current study when we associated the distractor with a highly concentrated quinine solution, the discrimination of perceptually similar stimuli was possible, while it was not when the distractor was associated with water.

In the context of the hypothesis that differential conditioning to color stimuli improves performance by recruiting attentional processes [15], the difference in performance reported in our study suggests that attentional processes are enhanced by the penalizing effect of the aversive quinine solution. This may result in an improvement of the bees' discrimination performance, when compared to a situation where the distractor penalty is low or non existent. Thus our new finding is likely to be of value for testing the hypothesis of attentional processes in insect brains [15]. In any case, our results go against the idea that the difference between two colors is an immutable property constrained by the visual machinery of the honeybee. Rather, they indicate that aversive reinforcements may modulate discrimination by acting on attentional processes.

Previous work, mainly on vertebrate models, has shown that pairing an aversive reinforcer with a distractor promotes an increase of the appetitive associative strength of the rewarded stimulus [16]. This interpretation is not mutually exclusive with that provided above. In differential conditioning, experience with a CS+ and a CS− leads to the formation of an appetitive (excitatory) and an aversive (inhibitory) memory trace, respectively. Choice results from the interaction between these two traces so that if the subjective intensity of one of the reinforcers, the appetitive or the aversive, overcomes that of the alternative reinforcer, choice would be biased towards the dominant CS. In our study, although the appetitive reinforce (1 M sucrose) remained constant on the CS+ for experiments, the presence of quinine on the CS− (distractor) may have enhanced the relative value of the appetitive reinforcer, thus promoting not only avoidance of the distractor, but also enhanced preference of the target CS+.

Our findings have several important implications for research on cognition using free-flying honeybees as a model. In conceptual terms, they underline the necessity to study attentional processes in the honeybee and to relate them to specific neural structures in the bee brain. Moreover, they raise the fundamental question of the real limits of the bees' visual capacities. Several studies that have reported negative results in terms of visual discrimination capabilities in bees [30]–[31] may be missing the fact that bees were not paying attention to the cues that they were supposed to learn in a given task. Thus, before concluding that bees are not capable of solving a certain visual discrimination, researchers should address the critical question of whether their experimental designs are able to push the cognitive capacities of bees to their limits. The example provided by the perceptual similar pair of colors in Fig. 3, which cannot be discriminated if the distractor is paired with water but which can be distinguished if it is paired with concentrated quinine solution underlines this point. Besides, using such a more efficient training may allow a more robust analysis of the cognitive mechanisms contributing to perception, by decreasing the amount of training necessary to tackle specific complex experimental questions. For example, recent studies which had use quinine had been successful in using bees to study processing of complex pictures such as human faces [7], [10], [32].

Interestingly, the enhancing effect of the aversive reinforcement was not evident when stimuli were perceptually distinguishable (Fig. 2). This lack of effect can be interpreted as the negative reinforcement not being necessary to solve an easy problem, i.e. performance without quinine already reached a high level of correct choices (Fig. 2). An alternative, although not mutually exclusive, explanation can be raised in term of speed-accuracy trade-off. For the discrimination of large color distances (a simple discrimination task) bees making relatively fast decisions easily choose the correct color with a very low rate of error. Thus, even if bees made decisions slower and more carefully when the cost of making an error was increased by adding quinine, it didn't significantly change accuracy. For example, a significant difference in accuracy between fast and slow bumblebees was consistently found only when colors were perceptually close [18]. In this case, bumblebees were fast when they solved simple tasks but when the task became more difficult some individuals decreased the speed by which they chose, thus leading to an increase of performance [18]. Such modulation of response time based on the perceptual difficulty of the task is well-known in humans [33]. Equivalent data are still missing for honeybees. Moreover, there may be differences in visual processing between different bee species [34]. Thus, measuring the time allocated for decision making by free-flying honeybees confronted with tasks of different difficulty may constitute an interesting research perspective for the future [35]–[36].

### Quinine solution as a negative reinforcement

The concentrations of quinine used in our work are far from being ecologically relevant, as they were highly concentrated. The use of these highly concentrated solutions is justified by the fact that we wanted to associate an intense negative experience with the CS− and that free-flying bees seem to be more tolerant than humans to intermediate concentration levels of bitter substances [37]. Deterrent secondary compounds such as alkaloids or phenolic compounds are naturally present in nectar or pollen flowers as a defense against herbivores, but their concentration levels never reach those used in our experiments. It has even been reported that at natural concentration levels in the nectar, these substances may be neutral or even attractive for honeybees [21]. However, when bees have the choice between two nectars or pollens, one with secondary compounds and another with less secondary compounds, they usually prefer the latter [38]–[40]. Thus, the use of unnatural, higher concentrations allows establishing an effective aversive reinforcement for our studies and uncovering in this way the real visual discrimination abilities of honeybees.

In concluding that the use of quinine improves learning performance, we need to specify that the experimental conditions in which quinine acts as a negative reinforcement are those provided by our work, i.e., free-flying bees subjected to visual discriminations. It therefore appears that the critical aspect of our experimental procedure is the fact that honeybees could free move and, more importantly, free express their avoidance of the quinine solution as a negative reinforcer.

This aspect may explain apparent contradictory results on the effect of substances like quinine on the behavior of bees in the laboratory (see [24] for review). In the laboratory, contrary to the experimental results described above, bees are harnessed in individual metal tubes, which is the common procedure to test their sucrose responsiveness and/or learning in olfactory conditioning using the proboscis extension reflex (see [41] for review). In these experimental conditions, harnessed bees do not show an aversion for even higher concentrations of quinine solution than that used in the current study [22], [25]. The same lack of aversion applied to a variety of substances that also taste extremely bitter to humans (salicine, amygdalin, caffeine, etc.) [22], [25]. Furthermore, harnessed bees imbibe large amounts (20 µL, one third of their crop capacity) of aversive solutions, including quinine solution, even if the imbibed solutions turn to be toxic and induce post-ingestional mortality [25]. In the case of studies on honeybee gustation in the laboratory (harnessed bees), recent results have suggested that the main effect produced by bitter substances is not a distasteful gustatory one, as suggested in our case, but rather a post-ingestional one, given that in all cases bees drank the aversive compounds without reluctance [25]. This difference with the current study may be due to the fact that in both experimental contexts, the capacity to express an active avoidance of the aversive reinforcement varies dramatically. When bees are in contention, the impossibility of movement may induce important changes in acceptance or rejection thresholds for gustatory compounds, making the bees more tolerant to substances that they would otherwise reject, even at the cost of their own death.

This hypothesis is plausible given that harnessed and free-flying bees exhibit striking differences in performances of other tasks such as color learning and discrimination. Experiments with free-flying bees have shown that the Δλ discrimination function (i.e. the function accounting for the bees' wavelength discrimination along their visual spectrum) varies depending on the region of the spectrum. It reaches extremely fine discrimination values of 4.5 nm for wavelengths at the intersection of photoreceptor sensitivity curves [42]. On the contrary, harnessed bees in the laboratory, which can be trained to associate a color with sucrose reward and which extend their proboscis to the learned color [43], have difficulties in learning this association and show very poor color discrimination abilities [44]. This difference may be motivational, as to learn colors in harnessed conditions it is necessary to cut the bees' antennae [43], [45]. This procedure substantially decreases the subjective value of sucrose as a reward [46], thus impairing learning. Therefore our data confirm that studying free-flying bees remains essential to approach the natural behavior and learning capabilities of this animal as a model for neuroscience.

Whilst our study shows that honeybees do have an ability to detect quinine solution, the physiological mechanisms by which they do this are still unclear. Our results support a peripheral detection via gustatory receptors (see Experiment 3), but so far, bitter receptors have not been found in electrophysiological experiments [22] nor in genomic analyses comparing honeybee gustatory receptor genes with those of the fruit fly *Drosophila melanogaster*. In the fruit fly, 68 gustatory receptor genes have been identified [47]–[50]. Two of these genes have been associated with bitter taste as they both respond to caffeine and are coexpressed in the same gustatory receptor neurons [51]–[52]. In the honeybee, the picture seems to be drastically different: only ten gustatory receptor genes were found [23] suggesting that the gustatory world of a bee might be considered as relatively poor. Among these receptors, two are similar to the trehalose (‘sweet’) receptor of flies, but none is similar to their ‘bitter’ receptors. In our experiments, the bees extended the proboscis before being repelled by the quinine solution, which leads us to hypothesise that they detect the presence of the aversive quinine solution via a gustatory input. The concrete mechanism by which this is achieved remains unknown, and thus warrants further investigation.

### Conclusion

The present study supports the hypothesis that aversion conditioning is important for understanding how and what animals perceive and learn. By using this form of conditioning coupled with appetitive conditioning in the framework of a differential conditioning procedure, it is possible to uncover discrimination capabilities that may remain otherwise unsuspected. We show that that what an insect sees and learns may be significantly affected by experience rather than being only deductible from its visual machinery. Further experiments studying visual discrimination capabilities of honeybees should consider using true negative reinforcements associated with the CS− in order to reveal what these insects can really perceive.

## Materials and Methods

### Experiment 1

Individual free-flying honeybees *Apis mellifera*, Linnaeus, from a single colony located 100 m from the test site were trained to collect 0.2 M sucrose solution from an artificial feeder. The feeder was located 10 m from a Y-maze (Fig. 1a) to which individually marked honeybees selected from the feeder were trained to collect 1 M sucrose solution [53]. Only one individual was present at a time in the Y-maze, which was covered by an ultraviolet-transparent Plexiglas ceiling. The maze was located on an outside table and illuminated by open daylight. The entrance of the maze led to a decision chamber, where the honeybee could choose between the two arms of the maze. Each arm was 40×20×20 cm (L×H×W). The back walls of the maze (20×20 cm) were placed at a distance of 15 cm from the decision chamber and were covered by a white reflecting UV background on which color targets were presented. Each color target consisted of a 7×7 cm square cut from a HKS-N paper (K+E Stuttgart, Stuttgart-Feuerbach, Germany). Targets were placed in the middle of their corresponding back wall (Fig. 1a). They therefore subtended a visual angle of 26° to the centre of the decision chamber and were thus large enough to recruit the chromatic pathways of the honeybee's visual system [53].

The reflectance spectra of the stimuli were measured with a spectrophotometer (Ocean Optics SD2000 with a DT1000 mini light source (200–1,100 nm) and R400-7 UV/VIS optical fibre). The perceptual distances between colors were calculated using the Color Opponent Coding (COC) space (Fig. 2a; [54]) and the hexagon color space (Fig. 2a; [55]). In both cases, for calculations we used the spectral sensitivities of the honeybee photoreceptors [56], a standard daylight function D65 [57] and the white background used in our experiments. Four colors were used in this experiment (HKS 8N, 26N, 44N and 54N, which appeared orange, pink, blue and green to humans respectively; see Fig. 2a). For each bee (N = 18) three of these colors were chosen as CS+, CS− and NS, respectively (see below). Thus, a combination of three colors was assigned to each bee among the 24 combinations possible (e.g. CS+: 8N, CS−: 26N and NS: 44N). All colors were easily distinguishable for bees as they were well separated from each other in both color spaces used to represent them *(color opponent coding space*: mean perceptual distance between stimuli ± s.e.m.  = 6.15±1.10 COC units; *hexagon color space*: 0.31±0.04 hexagon units).

During differential conditioning, the CS+ was rewarded with 1 M sucrose solution while the CS− was associated either with tap water (water group) or 60 mM quinine hydrochloride solution (quinine group) (N = 9 for each group). Bees in the quinine group and in the water group (see below) were matched with respect to colors used. Solutions were delivered by means of a transparent micropipette 6 mm in diameter located in the centre of each visual target.

Conditioning consisted of 15 training trials (i.e. 15 foraging bouts between the hive and the maze). The side of the rewarded stimulus was interchanged following a pseudorandom sequence to avoid positional (side) learning (i.e. the same stimulus was not presented more than twice on the same side). If the bee chose the rewarded stimulus CS+, it could drink sucrose solution *ad libitum*. If it chose the non-rewarded stimulus CS−, it was allowed to taste the water or quinine solution and then to fly to the alternative arm presenting the CS+ to find the sucrose. On each trial, only the first choice of the bee was recorded for statistical analysis.

Acquisition curves for both the quinine and the water group were obtained by computing the frequency of correct choices during 3 blocks of 5 trials each. After training, three transfer tests with fresh, non-rewarded stimuli were performed: a learning test presenting the CS+ vs. the CS−, an ‘appetitive’ test presenting the CS+ vs. a novel stimulus NS, and an ‘aversive’ test presenting the CS− vs. the NS. During the tests, contacts with the surface of the targets were counted for 45 s. The choice proportion for each of the two test stimuli was then calculated. Each test was done twice, interchanging the sides of the targets to control for side preferences. Refreshing trials with the reinforced CS+ and CS− were intermingled between the tests to ensure motivation for the subsequent tests. The sequence of appetitive and aversive tests was randomized between bees.

### Experiment 2

The set-up and procedure used in this experiment were similar to those of experiment 1 (Fig. 1a) except for the colors used for training, which, in this case, were perceptually similar. Colors used for training half of the bees (N = 8) were HKS-3N vs. 68N, which appeared yellow to a human observer (perceptual distance separating them: 1.58 COC units; 0.08 hexagon units); the other half of the bees (N = 8) was trained with HKS-43N vs. 47N, which appeared blue to a human observer (perceptual distance: 1.36 COC units; 0.08 hexagon units). CS+ and CS− were counterbalanced between bees within each group. After the 15-trial conditioning, a non-rewarded learning test with fresh CS+ and CS− stimuli was performed.

### Experiment 3

Experiment 3 consisted of two parts. In the first part, a Y-maze was used to train the bees in a discrimination task that was visually impossible as the identical color was presented as CS+ and CS− in the arms of the maze. For half of the bees (N = 4), the training stimulus was HKS-8N (orange to humans) whereas for the other half (N = 4) it was HKS-54N (green to humans) (Fig. 2a).

The procedure used to train the bees was the same as in experiments 1 and 2, except that reinforcers were not delivered in micropipettes located in the middle of the color stimuli but on white plastic discs (26 mm diameter, 4 mm thick) lying on a horizontal landing surface standing on a 10 cm pole placed 1 cm in front of each stimulus (Fig. 1b). Thus, in each arm of the maze, a landing platform in front of the visual stimulus offered the reinforcer in a plastic disc. Each disc presented a small hole (4 mm diameter, 2.5 mm depth) to hold the fluid (Fig. 1b). The landing platforms were used in order to record accurately feeding behavior with a video camera (see below).

Between trials the discs were cleaned with 10% ethanol solution to remove odor cues. During the first five trials one stimulus was rewarded with 35 µL of 1 M sucrose solution (‘target’) whilst the other stimulus contained no fluid (‘distractor’). In the next five trials, the ‘distractor’ stimulus was associated with 35 µL of 60 mM quinine hydrochloride; finally, in the last five trials, it was associated with 35 µL of tap water. For another group of bees, the sequence of the quinine – water trials was inverted.

During all trials a video camera (Canon MV920) was used to record the time spent by the imbibing the reinforcers (evaluated through proboscis extension time). To evaluate the volume (µL) fed using the drinking time (sec), we established a standard curve relating these two variables for 1 M sucrose solution. The sequence of presentation of different volumes (2, 5, 10, 20, 30, 40, 50, 60, 70 µL) was randomized. The viscosities of water and of the 60 mM quinine solution are lower than that of the 1 M sucrose solution. Thus, the standard curve obtained for sucrose 1 M was used to estimate the volume of water or quinine solution ingested.

In the second part of this experiment, we aimed at increasing the precision of our drinking measurements. We trained a new group of bees (N = 8) to land on a 20×20 cm UV transparent Plexiglas sheet housed within a 28.5×18.5×22.5 cm (L×H×W) box which had a UV transparent Plexiglas cover. To this end, individually-marked bees were allowed to collect 5–10 µL drops of a 1 M sucrose solution on the Plexiglas sheet until satiation. Only one bee was present at a time in the box. After imbibing the sucrose solution, the bee selected for the experiments was allowed to return to the hive and recordings began when it came back to the set-up.

The bee had to visit the box six times (i.e. six foraging bouts or trials). Each trial lasted 180 seconds, and at the end of it the bee was fed until satiation with 10 µL drops of 0.5 M sucrose placed on the Plexiglas sheet. After that, the bee was allowed to return to the hive. Within each trial the bee was allowed to collect small droplets (1 or 2 µL) of either sucrose (1 M), tap water or 60 mM quinine solution. In trials 1, 3 and 6 the bee was presented with 16 drops of either sucrose (4×1 µL and 4×2 µL) or water (4×1 µL and 4×2 µL), which were randomly arranged within a 4×4 grid on the Plexiglas sheet. In trials 2, 4 and 5 the bee was presented with 16 drops of either sucrose (4×1 µL and 4×2 µL) or quinine (4×1 µL and 4×2 µL), which were randomly arranged within the same 4×4 grid. Between trials the set-up was cleaned with 5% ethanol and afterwards with tap water. We recorded the number of drops of solution completely imbibed by a bee, and the proportion of solutions that were sampled.

### Statistical analysis

Data were checked for normality using the Shapiro-Wilk test and when necessary subjected to an arcsine transformation. Performance of balanced groups during acquisition was compared by means of a two-factorial ANOVA of repeated measurements in which the stimuli used constituted one factor and the negative reinforcement (water or quinine) the other factor. The dependent variable was the percentage of correct first choices of each individual bee in each block of 5 trials.

Performance during the tests was analyzed in terms of the proportion of correct choices per test (i.e. a single value per bee). A one-sample t-test was used to test the null hypothesis that the proportion of correct choices in the test considered was not different from a theoretical value of 50%. Comparison between groups was made using an independent two-sample t-test. The alpha level for statistical significance was 0.05.
